# Women's sexual health and contraceptive needs after a severe obstetric complication ("near-miss"): a cohort study in Burkina Faso

**DOI:** 10.1186/1742-4755-7-22

**Published:** 2010-08-27

**Authors:** Rasmané Ganaba, Tom Marshall, Issiaka Sombié, Rebecca F Baggaley, Thomas W Ouédraogo, Véronique Filippi

**Affiliations:** 1 Agence de Formation, de Recherche et d'Expertise en Santé pour l'Afrique (AFRICSanté), 01 BP 298 Bobo-Dioulasso, Burkina Faso; 2 Department of Epidemiology and Population Health, London School of Hygiene & Tropical Medicine (LSHTM), London, UK; 3 West African Health Organization (WAHO), Bobo-Dioulasso, Burkina Faso; 4 MRC Centre for Outbreak Analysis and Modelling, Department of Infectious Disease Epidemiology, Faculty of Medicine, Imperial College London, London, UK

## Abstract

**Background:**

Little is known about the reproductive health of women who survive obstetric complications in poor countries. Our aim was to determine how severe obstetric complications in Burkina Faso affect reproductive events in the first year postpartum.

**Methods:**

Data were collected from a prospective cohort of women who either experienced life threatening (near-miss) pregnancy-related complications or an uncomplicated childbirth, followed from the end of pregnancy to one year postpartum or post-abortum. Documented outcomes include menses resumption, sexual activity resumption, dyspareunia, uptake of contraceptives, unmet needs for contraception and women's reproductive intentions.

Participants were recruited in seven hospitals between December 2004 and March 2005 in six towns in Burkina Faso.

**Results:**

Reproductive events were associated with pregnancy outcome. The frequency of contraceptive use was low in all groups and the method used varied according to the presence or not of a live baby. The proportion with unmet need for contraception was high and varied according to the time since end of pregnancy. Desire for another pregnancy was highest among near-miss women with perinatal death or natural abortion. Women in the near-miss group with induced abortion, perinatal death and natural abortion had significantly higher odds of subsequent pregnancy. Unintended pregnancies were observed mainly in women in the near-miss group with live birth and the uncomplicated delivery group.

**Conclusions:**

Considering the potential deleterious impact (on health and socio-economic life) of new pregnancies in near-miss women, it is important to ensure family planning coverage includes those who survive a severe complication.

## Background

Several studies have described the magnitude and determinants of obstetric complications in poor countries [1-4], but there is little information on the consequences of these complications, particularly for women's sexual lives and their use of contraception. To date, a small number of studies conducted in high-resource countries have focused on the reproductive health consequences of "near-miss" obstetric complications, when the chance of survival of women has been particularly severely compromised. They show that these complications significantly influence women's sexual health, wellbeing and fertility [5,6]. However, these results may not be generalisable to low resource settings because of differences in health systems and cultural contexts.

Most of the available information on women's postpartum reproductive health in sub-Saharan Africa originates from cross-sectional studies [7-13] and a few prospective studies [14-17]. Women report a high prevalence of reproductive morbidity in the postpartum period (22-68%), in particular dyspareunia (painful sexual intercourse) and loss of sexual desire: 34% and 40%, respectively, at three months [16] and 14-19% and 8.5-26%, respectively, at six months [12,17]. A recent analysis of 35 African datasets from 22 countries showed that women who underwent emergency caesarean section have lower postpartum fertility than women with a vaginal delivery and that this could be explained by voluntary and involuntary factors [8].

Current World Health Organization recommendations are that women should wait until their youngest child reaches their second birthday before becoming pregnant again [18]. Contraceptive uptake in the postpartum period is generally low (< 40%) in most poor countries [7,13]. Barriers to family planning in these countries are well known and include difficult geographical access, poor method choice, inadequate visit schedules, high financial costs, low women's status, medical and legal restrictions, provider bias and fear of side effects [19]. Extended durations of postpartum abstinence characterize traditional postpartum regimes in sub-Saharan Africa [7,10,20,21], but this tradition is less practiced nowadays, especially among urban populations [7,10,12,14,17,20,22]. Demographic and Health Surveys (DHS) data from Ghana, Kenya and Zimbabwe have suggested that the combined effect of amenorrhoea (affected by the frequency and intensity of breastfeeding) and abstinence provides natural protection from pregnancy for an average of one year or more [7] which might also influence the perceived need for additional contraceptive use.

The objective of this paper is to assess whether there are differences in reproductive events in the year after the end of pregnancy in a cohort of women who either experienced severe pregnancy-related ("near-miss") morbidity or an uncomplicated childbirth. Among those with a severe complication, we also investigate whether live birth, natural abortion, induced abortion, and perinatal death influence reproductive health in the postpartum period in dissimilar ways. We examined two main hypotheses: women with a near-miss complication and a live birth were more likely to try to control their fertility compared to women with uncomplicated delivery; and near-miss women with a perinatal death and/or an early pregnancy loss were more likely to want to become pregnant again as soon as possible than near-miss women with a live birth.

## Methods

### Study design, recruitment and follow-up procedures

We use data from a prospective cohort study which took place in six towns of Burkina Faso (Ouagadougou, Bobo-Dioulasso, Tenkodogo, Dédougou, Nouna and Houndé) and their surrounding areas. The recruitment was carried out in seven hospitals between December 2004 and March 2005 by specially trained midwives and doctors. Our study was hospital-based due to the difficulty in accurately determining whether a woman has had a severe obstetric complication based on individual interviews alone [23-25].

Women were invited to participate when they were admitted to the hospitals' maternity units with the signs and symptoms of a near-miss complication associated with the end of a pregnancy and when they lived within 25 to 30 km of the hospitals. For each case of near-miss complication recruited, we enrolled two women with uncomplicated delivery as controls. The radius of 25-30 km was arbitrarily set to facilitate motorcycle access to the homes of all participating women and to ensure that the two groups of women lived in broadly similar socio-economic environments.

Our study documents women's reproductive lives from the end of pregnancy to one year postpartum or post-abortum. To ensure good follow-up rates, women were contacted by female lay interviewers before hospital discharge. After consent, they were accompanied to their homes in order to document their address in detail, using drawing and landmarks. During this time, a health provider (a doctor or midwife) prepared a summary of their medical records. Women were interviewed at home four times during the follow up period: within a week of discharge and at 3, 6 and 12 months after the end of pregnancy. Health examinations were also carried out at 6 and 12 months by eight gynaecologists and obstetricians, using standardised procedures agreed during a workshop they attended. Among other observations, this allowed the diagnosis of new pregnancies, by means of palpation (at 6 months) or urine pregnancy test (at 12 months, using the HCG Strip test (Clinotech Diagnostics, Inc. http://www.clinotech.com)), and the determination of the presence and type of female circumcision. Other health, social and economic consequences of obstetric complications were also documented [26,27].

The study protocol was approved by the ethics committees from the London School of Hygiene and Tropical Medicine, United Kingdom, and the Centre MURAZ, Burkina Faso.

### Case definitions for near-miss complications and uncomplicated childbirth

Women were classified as "near-miss" cases if they had indications of extreme clinical severity, such as signs of shock or organ failure. They included five categories of complications: severe distocia (uterine rupture and bandle ring); severe haemorrhage (leading to shock, emergency hysterectomy and/or blood transfusion and/or coagulopathy); severe infection (hyperthermia > 39°C, hypothermia < 35°C and/or a clear source of infection with clinical signs of shock); eclampsia/severe pre-eclampsia (blood pressure > 140/90 mmHg or increase in systolic blood pressure > 30 mmHg or diastolic blood pressure > 15 mmHg with at least one other sign of severity such as convulsion, coma, icterus, pulmonary edema, thrombocytopenia < 100,000 platelets); severe anaemia (haemoglobin level < 4 g/dl or 4-7 g/dl or palor with signs of shock or difficulty of breathing or blood transfusion).

Women suffering a near-miss event ("near-miss women") are categorized depending on their pregnancy outcome: those experiencing a live birth; those suffering a perinatal death before discharge; those with natural abortion including miscarriage or ectopic pregnancy; and those with induced abortion, defined according to WHO probabilistic criteria [28]. For some analyses, we also distinguished the experience of women with near-miss complications in association with a caesarean section as reported in medical records.

An uncomplicated childbirth was defined as taking place in a health facility, ending in a live birth at term and occurring vaginally, without complication in the third trimester and at the time the pregnancy ended. However, a woman with uncomplicated delivery may have had an episiotomy, forceps or vacuum extraction to facilitate the birth of her baby.

### Definitions of main outcomes

The questionnaire included questions on family planning and fertility similar to those used in Demographic and Health Surveys. The main outcomes of interest were: (i) return of menses; (ii) sexual activity resumption; (iii) uptake of family planning (assessed through reported use of contraception, modern or traditional, and description of methods and context surrounding their use/non use); (iv) women's desire for another baby; (v) subsequent pregnancy, assessed by clinical examination and/or immune test or the reported occurrence of a new delivery/pregnancy loss; (vi) unmet need for contraception, defined as the family planning needs of women with restarted menses (excluding women with hysterectomy) who resumed sex, but did not use contraceptives and wanted to delay (beyond one year) or limit childbearing; and (vii) dyspareunia.

### Statistical analysis

The statistical analysis was performed using STATA software (Version 9.2). Frequencies were calculated before comparing groups using multiple logistic regression models. The natural abortion and perinatal death groups were combined for the analysis. Thus, comparisons were performed between near-miss women with induced abortion, near-miss women with perinatal death or natural abortion, near-miss women with a live birth, and the uncomplicated delivery group. We also compared contraceptive use in women with c-section to that in women who delivered vaginally, by means of a logistic model. Logistic models were controlled for age group (15-19, 20-24, 25-29, 30-34, 35+ years), gravidity (1, 2-4, 5+ pregnancies), education (formal education, no education), marital status (single, married/living with a partner in monogamous union, married/living with a partner in polygamous union), and socio-economic status (wealth quintiles), as described elsewhere [26]. When comparing incidence of dyspareunia between groups, we controlled for age group, parity, female genital mutilation (FGM), and episiotomy.

## Results

We invited 1042 women and 1014 consented to take part in the study. Among those who consented, there were 74 near-miss women with perinatal death before discharge; 199 near-miss women with live births and 677 uncomplicated deliveries. Eighteen near-miss women with induced abortion (10 induced abortions and 8 probable induced abortions, according to WHO classification [28]); 28 miscarriages and 17 ectopic pregnancies were classified as near-miss women with natural abortion. One near-miss woman with abortion but without probabilistic criteria information, who was experiencing her third abortion and who stated that this was a miscarriage, was classified in the natural abortion group giving a total of 46. Among the near-miss women, 21 with perinatal death before discharge and 79 with a live birth were delivered by caesarean. One woman with uncomplicated delivery was reported as having delivered by caesarean and was excluded from the analysis. Altogether 90.1% of the women who consented completed the study. Loss to follow up was mainly due to migration outside the study area [26].

The mean age was not significantly different among the five groups but there were significant differences for education, marital status and wealth quintile [26]. Marginally significant differences were also observed for parity and gravidity (Table 1). At the exit interview, near-miss women with induced abortion reported having fewer live children (Table 1). Both near-miss women with induced abortion and those with natural abortion were marginally more likely to express a desire for more children compared with other groups. Proportion of women with FGM did not differ significantly between groups; but increased with age (data not shown). Frequency of 3^rd ^degree FGM (the most severe) was higher in groups with higher mean age.

**Table 1:** Characteristics of women's reproductive history by pregnancy outcome, as recorded in the hospital or the exit interview.

	Near-miss								Uncomplicated delivery		Stat Sig (p)
				
	Induced abortion		Natural abortion		Perinatal death		Live birth				
Age No of respondents	18		46		73		199		675		
No & % for age <20 years	6	33.3%	10	21.7%	12	16.4%	46	23.1%	127	18.8%	0.5
20-24	3	16.7%	11	23.9%	24	32.9%	61	30.7%	206	30.5%	
25-29	4	22.2%	8	17.4%	18	24.7%	37	18.6%	166	24.6%	
30-34	4	22.2%	9	19.6%	8	11.0%	28	14.1%	107	15.9%	
35 & over	1	5.6%	8	17.4%	11	15.1%	27	13.6%	68	10.2%	
Mean	24.4		26.7		25.8		25.3		25.6		
Parity (including this pregnancy) No of respondents	18		46		74		199		676		
No & % for parity 0-1 deliveries	13	72.2%	19	41.3%	34	45.9%	91	45.7%	252	37.3%	
2-4	3	16.7%	19	41.3%	26	35.1%	74	37.2%	306	45.3%	
5+	2	11.1%	8	17.4%	14	18.9%	34	17.1%	118	17.5%	0.06
Mean	1.50		2.33		2.45		2.67		2.68		
Gravidity (including this pregnancy) No of respondents	18		46		74		199		676		
No & % for gravidity 0-1 pregnancies	6	33.3%	11	23.9%	21	28.4%	82	41.2%	218	32.2%	
2-4	9	50.0%	21	45.7%	32	43.2%	77	38.7%	326	48.2%	
5+	3	16.7%	14	30.4%	21	28.4%	40	20.1%	132	19.5%	0.09
Mean	2.61		3.67		3.45		2.88		2.93		
Early pregnancy losses and stillbirths (excluding this pregnancy) No of respondents	18		46		74		199		674		
No & % for losses & stillbirth 0	16	88.9%	34	73.9%	53	71.6%	153	76.9%	514	76.3%	
1	2	11.1%	9	19.6%	14	18.9%	38	19.1%	132	19.6%	0.6
2+	0	0.0%	3	6.5%	7	9.5%	8	4.0%	28	4.2%	
Mean	0.11		0.41		0.41		0.28		0.29		
Children Alive at time of interview No of respondents	18		46		74		199		675		
No & % for children alive 0-1	14	77.8%	25	54.3%	41	55.4%	101	50.8%	272	40.3%	
2-4	3	16.7%	17	37.0%	24	32.4%	73	36.7%	324	48.0%	
5+	1	5.6%	4	8.7%	9	12.2%	25	12.6%	79	11.7%	0.003
Mean children alive	1.17		1.85		1.91		2.25		2.38		
Wish for (more) children No of respondents	18		46		73		198		668		
No & % desiring more children	16	88.9%	36	78.3%	49	67.1%	124	62.6%	450	67.4%	0.08
Gender balance of children No of respondents (only those with one or more children alive)	11		30		48		195		671		
No & % happy with balance	8	72.7%	20	66.7%	30	62.5%	143	73.3%	506	75.4%	0.3
Intention at exit interview to use a contraceptive method No of respondents	18		46		74		199		675		
No & % intending to use	15	83.3%	27	58.7%	48	64.9%	133	66.8%	465	68.9%	0.3
Female circumcision ^1^ No examined	13		41		57		141		523		
No & % with no excision	3	23.1%	4	9.8%	7	12.3%	24	17.0%	78	14.9%	0.5
excision at 1^st ^degree	1	7.7%	4	9.8%	2	3.5%	11	7.8%	43	8.2%	
at 2^nd ^degree	9	69.2%	27	65.9%	42	73.7%	96	68.1%	373	71.3%	
at 3^rd ^degree	0	0.0%	6	14.6%	6	10.5%	10	7.1%	29	5.5%	

^1 ^based on 12-month medical examination; excludes non-attenders

Définitions:

Excision at 1^st ^degree: excision of clitoris with partial or total removal of the labia minora.

Excision at 2^nd ^degree: excision of the prepuce, with or without excision of part or all of the clitoris.

Excision at 3^rd ^degree: excision of part or all of the external genitalia and stitching or narrowing of the vaginal opening (infibulation).

### Breastfeeding

Breastfeeding was practiced by the vast majority of women with a live birth,: ranging from 97.6% to 94.7% when asked at 3, 6 and 12 months postpartum in the near-miss group with live birth, and 99.5% to 98.2% in the uncomplicated delivery group (Table 2). However, the women in the near-miss group were significantly less likely to breastfeed their newborn than women in the uncomplicated delivery group (Table 3). Seven women did not breastfeed their baby since around the time of birth (two near-miss live birth, one near-miss with a neonatal death before discharge, and four uncomplicated deliveries). The medical records of these women suggest that knowledge of HIV status did not play a role in this, as they were not tested and did not appear to be suspected of being HIV positive.

**Table 2:** Characteristics of women's reproductive history over the year after discharge, by pregnancy outcome, as recorded during the interviews at 3, 6 and 12 months

	**3 months**				**6 months**				**12 months**			
	
	**Nearmiss**				**Nearmiss**				**Nearmiss**			
	**Induced abortion**	**PND & natural abortion**	**Live birth**	**Un-complicated delivery**	**Induced abortion**	**PND & natural abortion**	**Live birth**	**Un-complicated delivery**	**Induced abortion**	**PND & natural abortion**	**Live birth**	**Un-complicated delivery**
	
Infant feeding No of respondents	NA	NA	165	653	NA	NA	150	631	NA	NA	141	607
No & % breastfeeding	NA	NA	161 97.6%	650 99.5%	NA	NA	142 94.7%	626 99.2%	NA	NA	134 95.0%	596 98.2%
No & % with breastfeed difficulties	NA	NA	25 15.2%	87 13.3%	NA	NA	16 10.7%	69 11.0%	NA	NA	16 11.3%	51 8.4%
No & % bottle feeding	NA	NA	11 6.7%	38 5.8%	NA	NA	9 6.0%	25 4.0%	NA	NA	7 5.0%	20 3.3%
No % % other feeding	NA	NA	4 2.4%	17 2.6%	NA	NA	103 68.7%	448 71.1%	NA	NA	134 95.0%	580 95.6%
Return of menses Number of respondents	18	114	179	660	14	105	161	638	15	107	158	624
No & % Menses returned	16 88.9%	92 80.7%	46 25.7%	153 23.2%	14 100%	94 89.5%	77 47.8%	280 43.9%	15 100%	91 85.0%	100 63.3%	428 68.6%
Resumption of sexual relations Number of respondents	18	114	178	657	15	111	164	640	15	103	155	597
No & % Resumed sexual relations	8 44.4%	64 56.1%	44 24.7%	186 28.3%	11 73.3%	94 84.7%	88 53.7%	388 60.6%	12 80.0%	96 93.2%	106 68.4%	444 74.4%
With Dyspareunia Number of respondents (1)	8	64	44	186	10	86	82	375	1	8	23	88
No & % with Dyspareunia	1 12.5%	15 23.4%	12 27.3%	58 31.2%	2 20.0%	10 11.6%	13 15.9%	51 13.6%	1 100%	1 12.5%	3 13.0%	6 6.8%
Pregnant or delivered or experienced an early pregnancy loss Number of respondents	18	114	177	657	14	100	160	625	15	108	160	629
No & % stating they were pregnant or delivered	0 0.0%	1 0.9%	0 0.0%	0 0.0%	1 7.1%	17 17.0%	4 2.5%	3 0.5%	6 40.0%	44 40.7%	9 5.6%	22 3.5%
Desire for another baby Number of respondents	18	114	179	661	13	93	160	636	12	80	157	619
No & % expressing desire	17 94.4%	92 80.7%	113 74.3%	491 74.3%	11 84.6%	74 79.6%	119 74.4%	484 76.1%	12 100%	60 75.0%	112 71.3%	461 74.5%
Desired timing Number of respondents	18	114	175	646	13	92	161	634	12	80	157	618
No and % want baby within a year	3 16.7%	36 31.6%	6 3.4%	6 0.9%	5 38.5%	26 28.3%	1 0.6%	3 0.5%	4 33.3%	20 25.0%	5 3.2%	10 1.6%
Currently using contraception Number of respondents	18	113	179	658	12	100	161	635	12	80	157	616
No & % using contraception	5 27.8%	45 39.8%	44 24.6%	169 25.7%	2 16.7%	40 40.0%	58 36.0%	245 38.6%	4 33.3%	36 45.0%	65 41.4%	249 40.4%
Of which												
% using Pill	4 80.0%	18 40.0%	7 15.9%	25 14.8%	1 50.0%	18 45.0%	16 27.6%	49 20.0%	3 75.0%	9 25.0%	11 16.9%	67 26.9%
% using IUD	0 0.0%	1 2.2%	0 0.0%	1 0.6%	0 0.0%	1 2.5%	2 3.4%	4 1.6%	0 0.0%	1 2.8%	2 3.1%	5 2.0%
% using Injection/implant	0 0.0%	4 8.9%	7 15.9%	20 11.8%	0 0.0%	5 12.5%	9 15.5%	35 14.3%	0 0.0%	9 25.0%	12 18.5%	38 15.3%
% using Condom	1 20.0%	9 20.0%	15 34.1%	60 35.5%	1 50.0%	7 17.5%	20 34.5%	103 42.0%	1 25.0%	8 22.2%	18 27.7%	85 34.1%
% using Natural method	0 0.0%	12 26.7%	12 27.3%	51 30.2%	0 0.0%	4 10.0%	3 5.2%	30 12.2%	0 0.0%	3 8.3%	15 23.1%	23 9.2%
% stating breastfeeding	n/a	n/a	2 4.5%	24 14.2%	n/a	n/a	0 0.0%	5 2.0%	0 0.0%	0 0.0%	0 0.0%	2 0.8%

^(1) ^asked only of those who resumed sexual relationships.

NA - not applicable; PND - perinatal death.

**Table 3:** Adjusted odds ratios with 95% confidence intervals for reproductive health outcomes and desire for further children up to 12 months after pregnancy, comparing groups by pregnancy outcome

				**Adjusted odds ratios (95%CI)**					
	
	**3 months**			**6 months**			**12 months**		
	
	**Induced abortion vs uncomplicated delivery**	**NM PND & natural abortion vs NM live birth**	**NM live birth vs uncomplicated delivery**	**Induced abortion vs uncomplicated delivery**	**NM PND & natural abortion vs NM live birth**	**NM live birth vs uncomplicated delivery**	**Induced abortion vs uncomplicated delivery**	**NM PND & natural abortion vs NM live birth**	**NM live birth vs uncomplicated delivery**
	
Breastfeeding	NA	NA	0.067 (0.007 - 0.67)	NA	NA	0.12 (0.03 - 0.51)	NA	NA	0.27 (0.08 - 0.91)
Return of menses	58.4 (10.1 - 339)	24.9 (11.6 - 53.8)	1.21 (0.80 - 1.82)	100% in abortion group	26.1 (10.2 - 66.8)	1.18 (0.80 - 1.75)	100% in abortion group	6.93 (3.16 - 15.2)	0.74 (0.48 - 1.12)
Resumed sexual relations	11.8 (3.3 - 42.0)	7.55 (3.88 - 14.7)	0.86 (0.56 - 1.33)	9.41 (2.56 - 34.6)	14.1 (5.72 - 35.0)	0.82 (0.54 - 1.22)	3.79 (0.94 - 15.3)	18.7 (5.58 - 62.5)	0.79 (0.50 - 1.24)
Dyspareunia	1.41 (0.09 - 22.8)	1.76 (0.47 - 6.54)	0.66 (0.27 - 1.61)	3.30 (0.39 - 27.6)	0.92 (0.30 - 2.88)	1.20 (0.59 - 2.46)	-^a^	-^a^	-^a^
Subsequent pregnancy	-^a^	-^a^	-^a^	41.67 (1.93 - 1000)	6.21 (1.91 - 20.41)	7.41 (1.48 - 37.04)	31.25 (6.49 - 142.86)	10.75 (4.65 - 24.39)	2.09 (0.91 - 4.78)
Use of contraception	3.11 (0.76 - 12.7)	3.05 (1.67 - 5.58)	1.26 (0.81 - 1.97)	0.94 (0.18 - 4.83)	1.67 (0.90 - 3.08)	1.01 (0.65 - 1.55)	2.17 (0.50 - 9.40)	1.69 (0.88 - 3.23)	1.27 (0.83 - 1.94)
Desires another baby	5.63 (0.61 - 52.0)	2.42 (1.09 - 5.37)	0.96 (0.58 - 1.60)	1.50 (0.22 - 10.2)	3.49 (1.33 - 9.13)	0.84 (0.49 - 1.44)	0.70 (0.31 - 1.59)	3.74 (1.43 - 9.74)	0.74 (0.42 - 1.30)
Desired timing of next pregnancy: within one year vs 2 to 5 years	Very large	15.8 (5.49 - 45.6)	4.17 (1.09 - 16.0)	-^a^	Very large	1.22 (0.08 - 18.2)	Very large	21.0 (4.75 - 92.9)	1.12 (0.32 - 3.93)

a) the group contrast was dropped from the regression model.

NA - not applicable; NM - near-miss; PND - perinatal death.

### Resumption of menses and sexual activity

At the three-month interview, more than 80% of near-miss women with induced abortion and near-miss women with perinatal death or natural abortion reported the return of monthly bleeding compared to 25% in the groups with a live birth (Table 2). At six and 12 months, the percentages of menstrual resumption increased in all the groups, but most substantially in the near-miss with live birth and uncomplicated delivery groups, reaching 63-69% (Table 2). Among the near-miss women with perinatal death or natural abortion, 17 never reported menses resumption; amongst those, 11 were pregnant and one woman had undergone a hysterectomy. One woman with hysterectomy recorded in her medical records reported the return of menses. This woman may have had a sub-total hysterectomy.

Near-miss women with induced abortion and those with perinatal death or natural abortion resumed sexual intercourse earlier than other groups. At three months, 44% and 56% reported that they had resumed intercourse respectively, compared to 25% for the near-miss women with a live birth and 28% for the women with uncomplicated delivery (Table 2). Frequencies of resumption increased in all groups at six and 12 months; however, by 12 months, one third of near-miss women with a live birth and a quarter of women with uncomplicated delivery still had not resumed sex. Adjusting for confounders, intercourse resumption was statistically more frequent in the near-miss group with induced abortion than in the uncomplicated delivery group and was more frequent in the near-miss group with perinatal death or natural abortion compared with the near-miss group with a live birth (Table 3).

Reasons given for delays in resumption of sexual activities centred mostly on health concerns, but reasons also varied according to the pregnancy outcome. For example, the infancy of the baby was a frequent reason given by near-miss women with a live birth and women with uncomplicated delivery (data not shown). No perceived need and partner's refusal were also reasons given by women in both near-miss groups. Dyspareunia was frequent, particularly at three months, in the group with uncomplicated delivery and in the near-miss group with a live birth (Table 2), but no statistical difference was observed between groups (Table 3).

### Family planning

**Figure 1:**
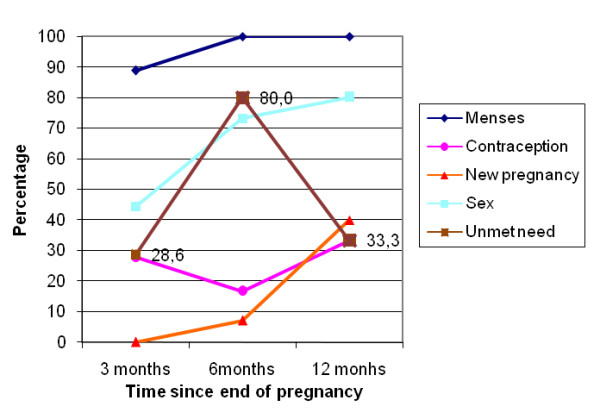
**Menses and sex resumption, contraception and unmet needs, new pregnancies in Burkina Faso in near-miss women with induced abortion**.

**Figure 2:**
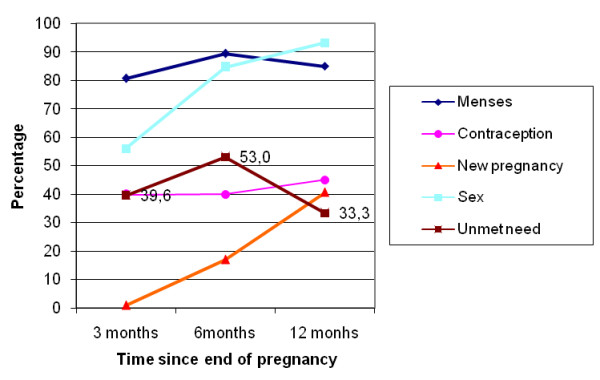
**Menses and sex resumption, contraception and unmet needs, new pregnancies in Burkina Faso in near-miss women with perinatal death or natural abortion**.

**Figure 3:**
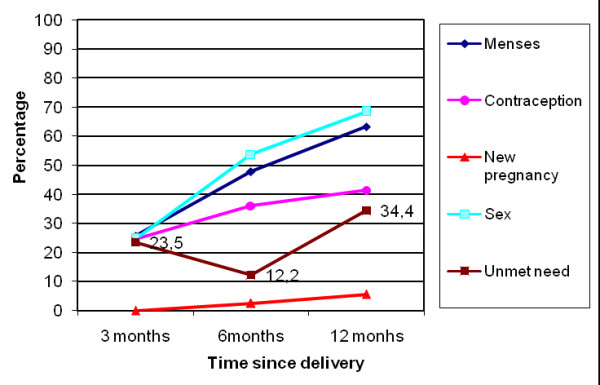
**Menses and sex resumption, contraception and unmet needs, new pregnancies in Burkina Faso in near-miss women with live birth**.

**Figure 4:**
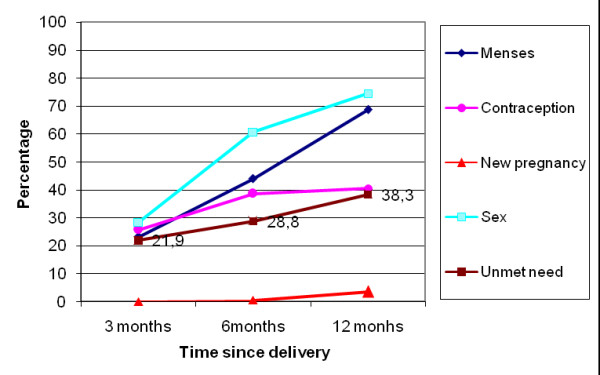
**Menses and sex resumption, contraception and unmet needs, new pregnancies in Burkina Faso in women with uncomplicated delivery**.

At the exit interviews, proportions of women in each group declaring intent to use a family planning method varied from 59% to 84% (Table 1). However, during subsequent interviews, the frequencies of contraceptive use were lower (Table 2, 25% to 40% at three months). Frequencies of contraceptive use increased slightly, from three to 12 months, in women with a live birth (in near-miss and uncomplicated delivery groups) but did not vary much in the other groups. Women frequently stated that their husbands knew about their contraceptive use (93% to 97%, according to pregnancy outcome group and interview). At three months, women in the near-miss group with perinatal death or natural abortion were significantly more likely to use contraceptive methods than women in the near-miss group with a live birth, but otherwise there were no statistical significant differences between groups (Table 3). The unmet need for contraception followed two general patterns: (i) in the near-miss group with induced abortion (Figure 1) and the near-miss group with perinatal death or natural abortion (Figure 2) there was a large increase at six month followed by a decrease at 12 months; (ii) in the near-miss group with live birth (Figure 3) and in the uncomplicated delivery group (Figure 4) the unmet needs of family planning increased from three to 12 months.

The great majority of near-miss women who delivered by caesarean and who resumed sexual intercourse by three months practiced contraception (83%). This proportion decreased at six months (66%) and remained stable (67% at 12 months). Women who delivered by caesarean with sex resumption were more likely to use contraception than women who delivered vaginally (adjusted odds ratio (OR), (95%CI): 5.0 (1.4-18.5) at three months, 5.6 (1.5-20.4) at six months, and 2.1 (1.1-3.9) at 12 months).

The principal contraceptive methods used were pills, condoms and natural family planning methods including withdrawal, followed by long-term methods (injection and implants) (Table 2). Contraceptive method differed by group (Table 2). While pills were preferred by women without a live birth, condoms were more frequently used by near-miss women with live birth and women with uncomplicated delivery.

Women gave a variety of reasons for using contraception including needing to rest/recover before a new pregnancy; fear of a new pregnancy; not wanting another baby; medical advice; and the current baby still being very young. The main reasons for not using contraception were: not yet having resumed sexual relations; menstrual cycle not re-established; factors associated with methods (cost, secondary effects); no knowledge of contraceptive methods; desire to become pregnant; and still seeking the husband's agreement.

### Reproductive intentions

Most women (71% to 100% by group and interview) wished for a new baby (Table 2). Near-miss women with perinatal death or natural abortion were significantly more likely to wish for a new baby than near-miss women with a live birth (Table 3). A statistically significant difference was also observed between groups regarding the ideal timing for the next pregnancy. Near-miss women with induced abortion and near-miss women with perinatal death or natural abortion were more likely to wish for a new baby within the next 12 months compared to women with uncomplicated delivery and near-miss women with live birth, respectively, of whom most would prefer another child in two to five years (Table 3). It was also observed, only at three months, that near-miss women with live birth were more likely to wish for a new baby within the next 12 months than women with uncomplicated delivery.

### New pregnancies

A total of 81 women experienced a new pregnancy, giving the following percentages by groups: 40.0% among near-miss women with induced abortion, 40.7% among near-miss women with perinatal death or natural abortion, 5.6% among near-miss women with live birth and 3.5% for women with uncomplicated delivery (Table 2). The near-miss groups with induced abortion and with perinatal death or natural abortion had significantly increased odds of new pregnancies compared with the groups with a live birth (Table 3). We also observed a significantly increased odds at six months in the near-miss live birth group when compared to the group with uncomplicated delivery.

According to the women's statements, 29.7%, 100%, and 57.1% of the new pregnancies in the near-miss group with perinatal death or natural abortion, the near-miss group with live birth, and the uncomplicated delivery group, were unplanned. One fifth (20.5%) of the new pregnancies reported by the near-miss women with perinatal death or natural abortion ended in an early pregnancy loss. For the other groups, the frequencies of new pregnancies were very low and therefore do not allow this breakdown. Among the women who were pregnant, those who had been exposed to a near-miss event with perinatal death or natural abortion were significantly more likely to experience an early pregnancy loss within 12 months post-pregnancy than near-miss women with a live birth (OR 19.7 (2.0, 194.1)).

Among the 81 newly pregnant women, 66.7% of near-miss with induced abortion, 34.1% of near-miss with perinatal death or natural abortion, 55.6% of near-miss with live birth and 72.7% of women with an uncomplicated delivery reported using contraception at three, six and/or twelve months. Five of the newly pregnant women had delivered the index pregnancy by caesarean section.

## Discussion

In our study, women who had a live baby are similar with regards to fertility intentions, contraceptive use and unmet contraceptive need, independent of their near-miss status. The near-miss experience did not influence their desire to have more children soon or their ability to use contraception. The loss of a baby or a pregnancy, intentional or no, appears to alter women's sexual health, reproductive intentions and pregnancy rates in the postpartum or post-abortum period compared to women with live babies. These differences relate mostly to the rapid return of menses, earlier resumption of sexual intercourse, increased desire for another baby (and at an earlier timepoint), and the incidence of new pregnancy. There was little difference between the groups in terms of contraceptive use and unmet need for contraception (which was substantial, at around 30-40%) and none with respect to dyspareunia. Although rates of breastfeeding were extremely high in our sample, women who were near-miss with live births were significantly less likely to breastfeed than women with an uncomplicated delivery. This finding has some importance, given the well known benefits that breastfeeding offers for the health of infants [29], and in view of other findings from our research project which show a higher mortality among infants of near-miss women [26].

### Strengths and limitations

The cohort design and rigorous case definitions for near-miss complications form the main strengths of this study, in addition to the very good follow up rate and the range of instruments and measurements that were used. The study was conducted in a low resource setting whereas the majority of previous research on the consequences of near-miss obstetric complications has been conducted in high- or middle-income countries, where life threatening events are rare in pregnancy and childbirth.

The most notable limitation of our study is that the sample is not fully representative of the childbirth experience in Burkina Faso because women were recruited in hospitals. This is particularly the case for the study participants with uncomplicated childbirth, who will have had a higher socioeconomic status than the other women. The sample is also mostly urban. The relatively small numbers for groups of women with near-miss complications do not allow sufficient precision around some of the estimates. In addition, the timing of our interviews might not have always been optimal. For example, no significant difference was found between groups in reporting dyspareunia. As the question referred to the last time the woman had sex, the result may have been different if the question had referred to the first time the women resumed sex after the end of their pregnancy. Finally, an ideal comparison group for near-miss women with induced abortion would have been induced abortion without complication. However, it is not possible to identify this group in Burkina Faso in view of the legal restrictions attached to the provision of induced abortion.

### Contraceptive use

Women reported an overall frequency of contraceptive use comparable to that reported in the 2003 DHS survey for 15-49 year old women in urban areas of Burkina Faso (34%) but higher than the value reported for women living in rural areas (10%) [30], with pills, condoms and contraceptive injection being the principal methods used. The types of contraceptive used varied considerably between groups, with women in stillbirth and early pregnancy loss groups more likely to use pills while women in the live birth groups used condoms. This difference is to be expected, since recommendations for type of contraceptive use depend on, among other conditions, breastfeeding status of women [31].

The majority of women were not using contraception (Figure 1, 2, 3, 4), mainly because they had not resumed sex (for women with a live birth) or they had resumed sex but wished for a new pregnancy (near-miss without a live birth). However, as was reported for 2003 by DHS [30], the unmet need for contraception is substantial, increasing in all groups from three to 12 months post-pregnancy, and explains the large number of reported unplanned pregnancies, mainly in women with a live birth. Similarly high levels of unmet need for contraception have been reported in other sub-Saharan countries [13,14,32]. However, our study disappointingly shows that even women who are users of maternity services experience a high level of unmet need in Burkina Faso. Current recommendations for improving uptake of contraceptive use include the provision of modern contraceptive methods to recently delivered women who want these before they leave hospital. Studies conducted by the Population Council, in particular in Nicaragua, have shown that the broader the choice of contraceptive type offered before hospital discharge, the higher the impact on uptake of postpartum contraception [33].

Our study found that substantial proportions of women who were using contraception (modern or traditional) became pregnant during the one year follow-up, in contradiction with their initial declaration that they wished for a new pregnancy two to five years later. Hubacher et al. (2008) estimated that every year in sub-Saharan Africa, approximately 14 million unintended pregnancies occur, mainly because of poor use of contraceptive methods [34]. There are several possible explanations for the lower use of contraception by women compared to their initial intention, although none are entirely satisfactory on their own. There may be social desirability bias, with women reporting what they expect study organisers want to hear; they may have simply changed their mind; or they may have had problems accessing family planning services [13]. Similar discrepancies between intention and subsequent use of contraception have been reported in several countries by Ross and Winfrey [13]. In addition, in our sub-sample of near-miss women, their initial memories of traumatic deliveries may have faded over time, and women were simply ready again to become pregnant or under pressure to do so by their partners or other family members [27].

### Desire for another child

Near-miss women with perinatal death or natural abortion are more likely to wish for a new baby, and this within 12 months, than women with uncomplicated delivery or near-miss women with a live birth. They resumed intercourse earlier and, as they were no more likely to use contraception at six and 12 months, they experienced new pregnancies more frequently. About 40% of these women were pregnant at 12 months and for three-quarters of these women, the new pregnancies were planned. High pregnancy rates have been reported elsewhere within a year after stillbirth in both a high income (UK) [35] and low income setting country [36]. Turton et al found that women who became pregnant relatively quickly after a stillbirth were also more likely to suffer mental health problems during their new pregnancy than women with a longer interval between pregnancies [37]. This has important implications for family planning programmes, in particular as a quarter of the women with stillbirth in our study reported their new pregnancy as unplanned. Women who have experienced a stillbirth get pregnant again for a range of personal reasons [37], and in this African setting with high fertility, there are considerable cultural and social pressures to have many children, as a woman's value is often defined by the number of children she has [38].

Women with a near-miss and induced abortion fell pregnant again quickly (Figure 1). This finding is more unexpected. Other studies have found that these women are usually very likely to use contraception [39,40]. For example, in Tanzania, 90% of women with an unsafe abortion accepted contraception at discharge and 86% were still using contraception at six months postpartum [40]. However, this study provided active contraceptive counselling and services which we did not make available in our observational study.

It is likely that there is some misclassification between the near-miss induced abortion and the near-miss natural abortion group in view of the probabilistic criteria that we used. This is important as women with spontaneous abortion are more likely to desire another pregnancy quickly. Nevertheless, other contextual factors may probably explain this finding, such as lack of contraceptives or access to good quality services when required, or the reliance on traditional methods, in particular because half of the women with termination in our sample were single. Women in Burkina Faso, as for women in many other countries, suffer from the lack of priority given by the government and donors for the provision of adequate family planning services [41]. More qualitative research is also required to understand the process of women's decision making post-termination in this high fertility African context.

Low pregnancy rates were observed in the two groups with live birth. In women with proven fertility, no complications of delivery, who have regular sex and are menstruating while not using any method of contraception, one would expect a 30% conception rate per month or about 50% per three months [42]. It is thus surprising that the overall pregnancy rate was so low at 12 months for the uncomplicated delivery group. They may not have been having sex frequently, or may have used natural family planning methods that were not identified or reported. The frequency of sexual intercourse is often lower in the first year postpartum than during the pre-pregnancy period and this is probably part of the explanation [43].

## Implications and conclusions

It is important to improve the activities and accessibility of family planning programmes in our setting in Burkina Faso, considering the high levels of unmet need for contraception and the high frequencies of unintended pregnancies among women using contraception. Given the limited resources, it may be important to prioritise women needing postpartum contraception. Women who have a near-miss complication will have a high risk pregnancy in their next pregnancy. This can lead to severe economic hardship for them and their family if they have to access medical care and treatment [27]. Postpartum family planning programmes should target near-miss women and meet their needs for contraception.

## Competing interests

The authors declare that they have no competing interests.

## Authors' contributions

VF was the lead investigator for this study. All authors (except RFB) participated in the design of the data collection instruments and the fieldwork which was coordinated by RG and IS. TM and RG analyzed the quantitative data. RG wrote the first draft of the paper with specific inputs from VF, and revised subsequent drafts. All authors contributed to the interpretation of findings and to the writing. All authors read and approved the final manuscript.
